# Supplementary material for: The RHIM of the Immune Adaptor Protein TRIF Forms Hybrid Amyloids with Other Necroptosis-Associated Proteins
Source: Molecules. 2022 May 24;27(11):3382. doi: 10.3390/molecules27113382 (PMC9182532; doi:10.3390/molecules27113382)
Supplement: Supplementary file 1 [file molecules-27-03382-s001.zip › molecules-1697235-supplementary.pdf]

molecules

MDPI

Article

The RHIM of the Immune Adaptor Protein TRIF Forms Hybrid Amyloids with Other Necroptosis-Associated Proteins

Max O. D. G. Baker [1], Nirukshan Shanmugam [2], Chi L. L. Pham [3], Sarah R. Ball [4], Emma Sierecki [5], Yann Gambin [6], Megan Steain [7] and Margaret Sunde [8,*]

Citation: Baker, M.O.D.G.; Shanmugam, N.; Pham, C.L.L.; Ball, S.R.; Sierecki, E.; Gambin, Y.; Steain, M.; Sunde, M. The RHIM of the Immune Adaptor Protein TRIF Forms Hybrid Amyloids with Other Necroptosis-Associated Proteins. *Molecules* **2022**, *27*, 3382. https://doi.org/10.3390/molecules27113382.

Academic Editors: Kunihiro Kuwajima, Yuko Okamoto, Tuomas Knowles and Michele Vendruscolo

Received: 9 April 2022
Accepted: 23 May 2022
Published: 24 May 2022

Publisher's Note: MDPI stays neutral with regard to jurisdictional claims in published maps and institutional affiliations.

Copyright: © 2022 by the authors. Submitted for possible open access publication under the terms and conditions of the Creative Commons Attribution (CC BY) license (https://creativecommons.org/licenses/by/4.0/).

Supplementary Figure 1.

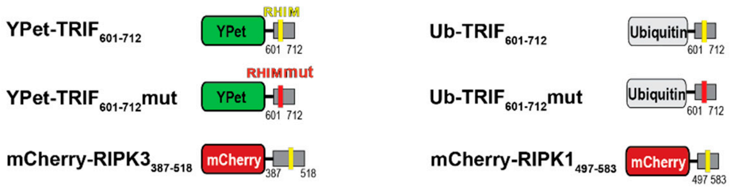

Figure S1. Schematic representation of fusion protein constructs used in this study**.** RHIM-containing protein domains of the indicated length, with wild type or RHIMmut AAAA core tetrad sequence, were expressed with a His-tagged ubiquitin or His-tagged fluorescent partner domain.

Supplementary Figure 2.

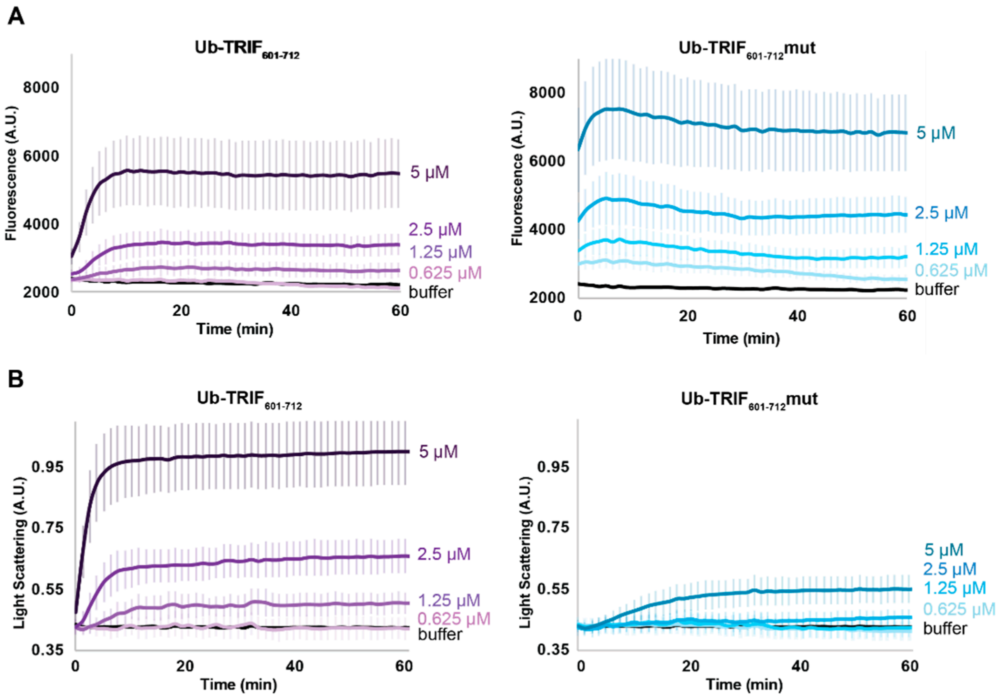

Figure S2. Concentration-dependence of assembly formation by Ub-TRIF RHIM constructs. In all cases, error bars indicate one standard deviation. **A.** Concentration dependence of ThT fluorescence over time for Ub-TRIF$_{601–712}$ and Ub-TRIF$_{601–712}$mut. Concentrations used are 0.625–5 µM, as indicated. **B.** Concentration dependence of assembly size over time for Ub-TRIF$_{601–712}$ and Ub-TRIF$_{601–712}$mut as determined by static light scattering. Concentrations used are 0.625–5 µM, as indicated.

Supplementary Figure 3.

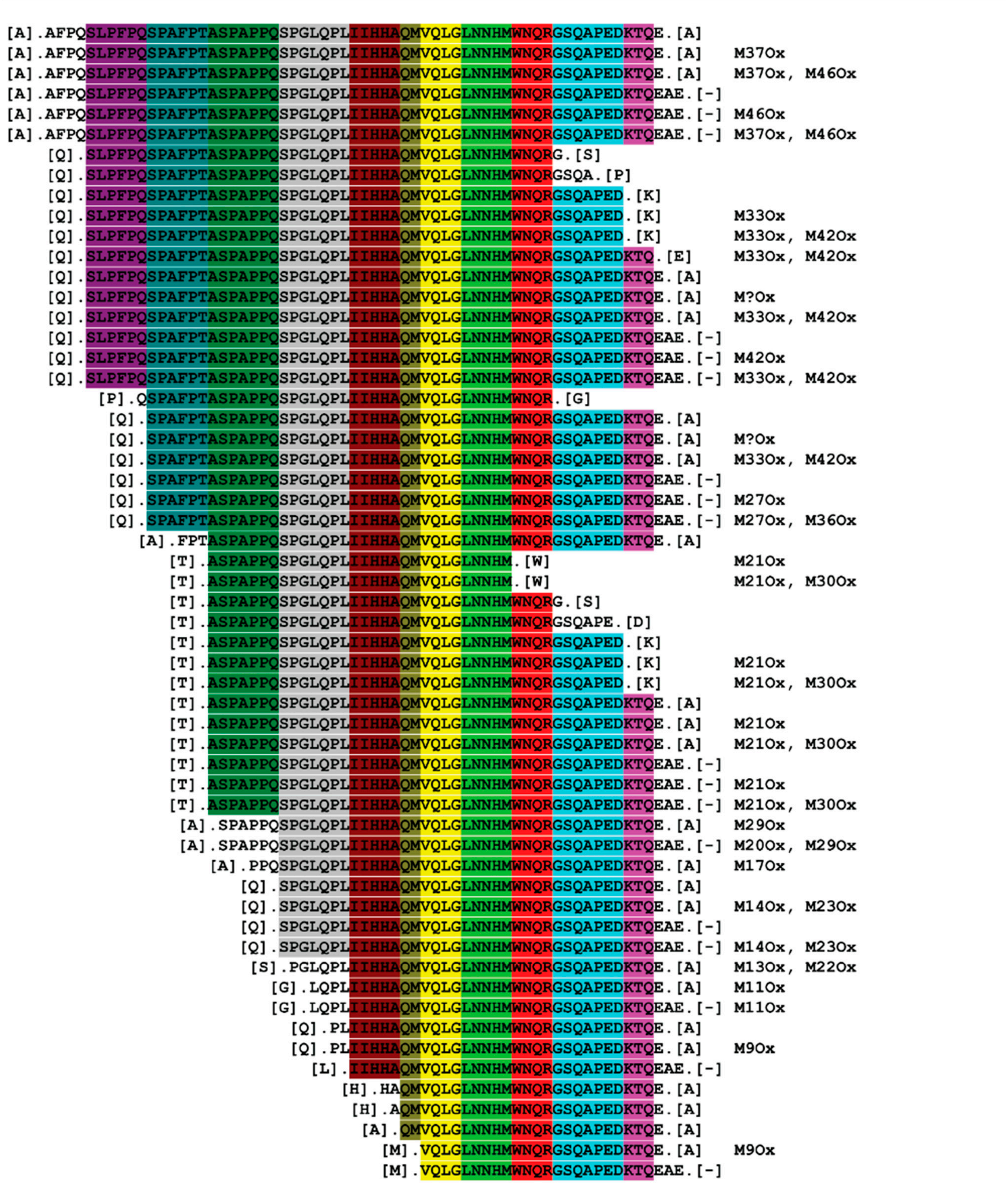

Figure S3. Complete list of sequences identified by mass spectrometry from the YPet-TRIF$_{601\text{-}712}$ RHIM core. Alignment of peptides identified by mass spectrometry, including posttranslational modifications. The oxidation of methionine likely resulted from the process of electrospray ionisation during mass spectrometry experiments. Residue series are coloured according to their prevalence.

Supplementary Figure 4.

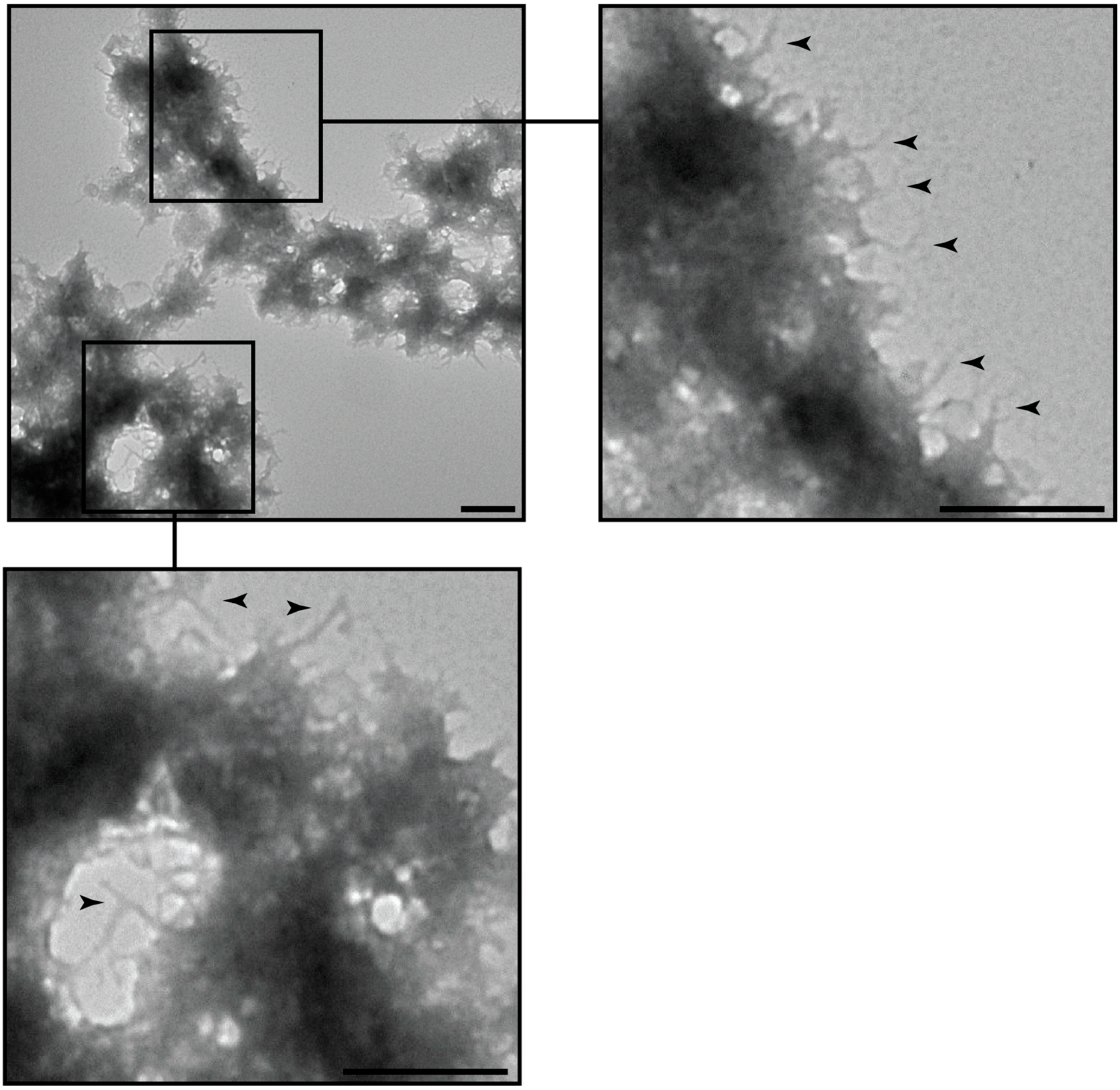

Figure S4. Morphological detail of TRIF-RIPK3 hetero-assemblies analysed by negative stain transmission electron microscopy. TRIF and RIPK3 form dense fibrillar bundles or clusters when co-assembled at 1 μM and individual fibrils emanate from the surface of the clusters. Boxed areas indicate zoom regions of the larger image. Black arrowheads indicate fibrillar material visible within or extending from the dense clusters. All scale bars represent 500 nm.

Supplementary Figure 5.

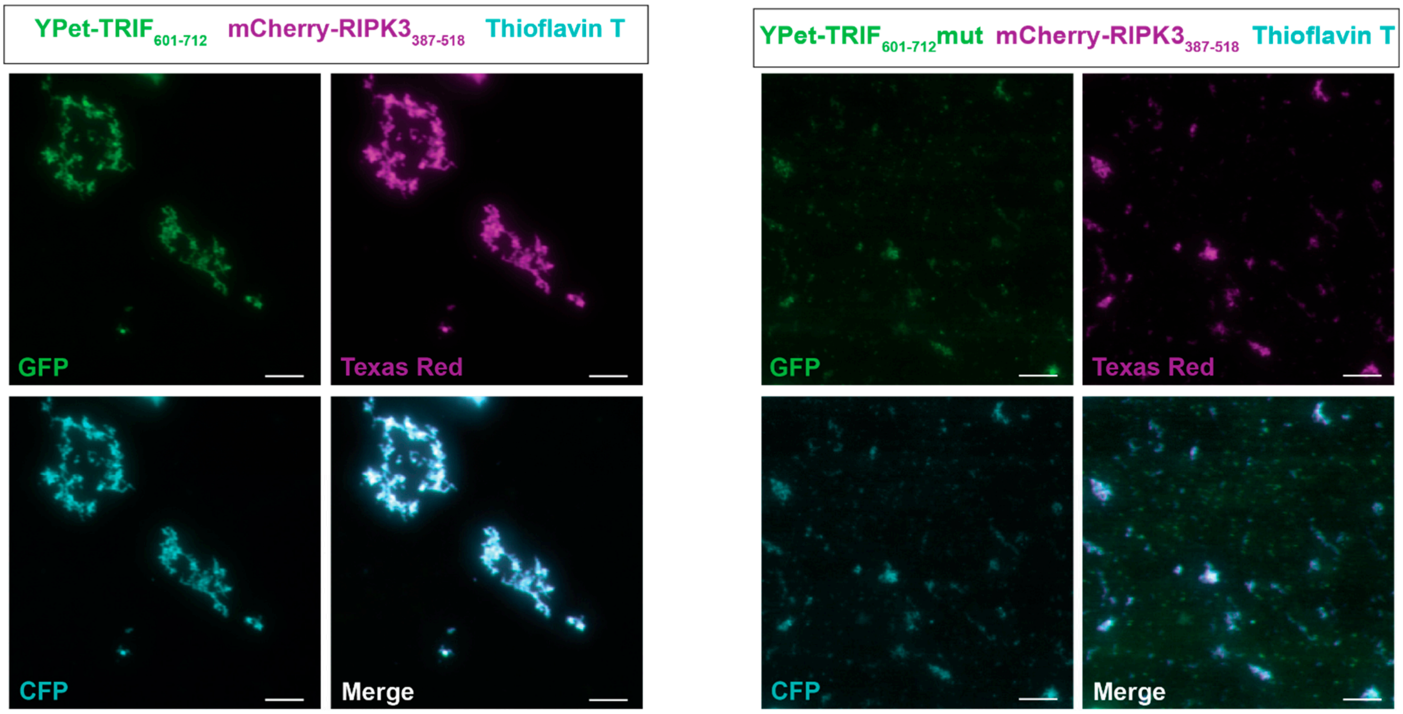

Figure S5. Widefield fluorescence microscopy of TRIF-RIPK3 hetero-assemblies co-stained with ThT. RHIM-containing proteins (as indicated above) from urea-containing monomeric stocks were diluted to 2.5 μM in assembly-permissive buffer containing 40 μM ThT. Protein assemblies were imaged using a Cytation 3 Imager (BioTek). CFP, GFP and Texas Red filter sets were used to detect ThT fluorescence, YPet and mCherry respectively. Scale bars indicate 20 μm.
